# Time since Onset of Disease and Individual Clinical Markers Associate with Transcriptional Changes in Uncomplicated Dengue

**DOI:** 10.1371/journal.pntd.0003522

**Published:** 2015-03-13

**Authors:** Cornelia A. M. van de Weg, Henk-Jan van den Ham, Maarten A. Bijl, Fatih Anfasa, Fatiha Zaaraoui-Boutahar, Beti E. Dewi, Leonard Nainggolan, Wilfred F. J. van IJcken, Albert D. M. E. Osterhaus, Byron E. E. Martina, Eric C. M. van Gorp, Arno C. Andeweg

**Affiliations:** 1 Department of Viroscience, Erasmus Medical Center, Rotterdam, The Netherlands; 2 Department of Internal Medicine, Faculty of Medicine, University of Indonesia, Jakarta, Indonesia; 3 Department of Microbiology, Faculty of Medicine, University of Indonesia, Jakarta, Indonesia; 4 Department of Biomics, Erasmus Medical Center, Rotterdam, The Netherlands; University of North Carolina School of Medicine, Chapel Hill, NC, UNITED STATES

## Abstract

**Background:**

Dengue virus (DENV) infection causes viral haemorrhagic fever that is characterized by extensive activation of the immune system. The aim of this study is to investigate the kinetics of the transcriptome signature changes during the course of disease and the association of genes in these signatures with clinical parameters.

**Methodology/Principle Findings:**

Sequential whole blood samples from DENV infected patients in Jakarta were profiled using affymetrix microarrays, which were analysed using principal component analysis, limma, gene set analysis, and weighted gene co-expression network analysis. We show that time since onset of disease, but not diagnosis, has a large impact on the blood transcriptome of patients with non-severe dengue. Clinical diagnosis (according to the WHO classification) does not associate with differential gene expression. Network analysis however, indicated that the clinical markers platelet count, fibrinogen, albumin, IV fluid distributed per day and liver enzymes SGOT and SGPT strongly correlate with gene modules that are enriched for genes involved in the immune response. Overall, we see a shift in the transcriptome from immunity and inflammation to repair and recovery during the course of a DENV infection.

**Conclusions/Significance:**

Time since onset of disease associates with the shift in transcriptome signatures from immunity and inflammation to cell cycle and repair mechanisms in patients with non-severe dengue. The strong association of time with blood transcriptome changes hampers both the discovery as well as the potential application of biomarkers in dengue. However, we identified gene expression modules that associate with key clinical parameters of dengue that reflect the systemic activity of disease during the course of infection. The expression level of these gene modules may support earlier detection of disease progression as well as clinical management of dengue.

## Author Summary

An acute dengue virus infection usually starts with a febrile disease phase that can progress to severe disease around the time fever abates (defervescence). Here we study dengue patients that were included very early after the onset of disease and carefully monitored in a longitudinal cohort study. Our results show that time after the onset of disease has a major impact on the transcriptome profile of patients with dengue, which is confirmed by comparing our results with three other dengue studies that included patients at different disease stages. There is a gradual shift in transcriptome profile from an ‘immunity & inflammation’- to a ‘repair & recovery’ phenotype. Furthermore, the expression of gene network modules could be linked to specific clinical parameters of dengue virus infection. Platelet counts and the levels of fibrinogen and albumin are shown to be good markers for the activity and timing of the disease, reflecting relevant biological processes in the patient. In contrast, conventional WHO classification systems did not show any association with any of the 25 gene modules identified and only yielded a few differentially expressed genes. The expression level of gene modules specific for certain biological processes in dengue support earlier detection of progression to severe disease and may improve clinical management.

## Introduction

Dengue virus (DENV) infection is endemic in South-East Asia and has a large impact on society, both in terms of burden of disease as in economic costs [1]. DENV belongs to the Flaviviridae family and consists of at least four serotypes: DENV-1,-2,-3 and -4. DENV infection has been described as a triphasic disease in the 2009 WHO dengue case classification [2]. The disease starts with the febrile phase in which all patients suffer from fever and a flu-like disease with general symptoms, such as fever, myalgia, arthralgia, headaches, and retro-orbital pain. After 3–5 days, patients enter the critical phase of disease, characterized by resolution of fever. The majority of patients with non-severe dengue recover in this phase, but some patients develop severe symptoms, such as shock, haemorrhage, or organ impairment, and they are classified as having severe dengue. Typical for the development of severe disease in the critical phase of dengue is a rapid decrease in platelet count with a concomitant increase in haemo-concentration due to plasma leakage. The critical phase usually lasts 24–48 hours, after which patients enter the recovery phase.

To investigate the underlying biological processes involved in DENV pathogenesis, several studies have applied transcriptome profiling to cohorts of dengue patients [3–5]. Some studies report that the acute (febrile) phase of dengue is characterized by an increased expression of genes involved in immunity and inflammation [6,7]. In this phase, transcripts involved in the innate immune response, in particular interferon induced genes and complement, are highly upregulated [6,7]. Other studies report that the convalescent (critical/recovery) phase is characterized by increased abundance of transcripts involved in cell cycle and cell repair mechanisms [5,8]. For disease severity in the acute phase of dengue, it has been shown that interferon-induced genes have a lower expression in patients with dengue shock syndrome (DSS) compared to patients with uncomplicated dengue [4,6]. In contrast, genes induced by the activation of neutrophils showed an increased expression level in patients with DSS [3,9]. This suggests that lower levels of interferon lead to impaired viral clearance and increased activation of neutrophils results in enhanced immune activation, which could both contribute to the development of severe dengue.

In this study, we investigated the biology of dengue pathogenesis over time in a cohort of dengue patients from Jakarta, Indonesia, where dengue is endemic and incidence increases during the rainy season [2]. Using a transcriptomics approach, we studied gene expression patterns, focusing on the association with dengue-specific clinical markers over time. We quantify the overlap of our data with results from other studies, focussing in particular on the stage of dengue disease. Furthermore, we perform a network analysis that relates clinical parameters to gene modules, offering potential markers for disease activity.

## Methods

### Ethics

The research ethics committee of the Faculty of Medicine, University of Indonesia in Jakarta, Indonesia, approved this study. Patients were included after written informed consent. If patients were younger than 18 years written informed consent was obtained from the parent and/or legal guardian. Data and samples were anonymized with a study number.

### Clinical cohort

Between March and June 2010 all patients ≥ 14 years of age with a fever onset ≤ 48 hours before presentation and a clinical suspicion of dengue were recruited in community health centers (‘puskesmas’) in Jakarta, Indonesia. Blood was drawn and a NS1 antigen and IgM/IgG antibody rapid test (SD Dengue Duo, Standard Diagnostics, inc, Korea) was performed. If tested positive for NS1 and/or IgM, patients were admitted to the Cipto Mangunkusomo Hospital in Jakarta for seven days. The admission was only dependent on a positive outcome from the rapid test and not based on clinical disease severity. Clinical data were recorded daily with a standard case report form. Blood samples were collected on every other day (including the day of admission), both for clinical laboratory tests and for transcriptome profiling. At day 3, 5 and 7 of admission ultrasound examination was performed to investigate whether patients suffered from ascites and/or pleural effusion. In this study, day 0 refers to the day of admission unless stated otherwise. According to the inclusion criteria, onset of disease was less than 48 hours before admission. Patients were classified according to the 2009 WHO dengue case classification [2]. Briefly, patients with fever and general symptoms were classified as non-severe dengue without warning signs (WS-). Patients with one of the following warning signs were classified as non-severe dengue with warning signs (WS+): abdominal pain, vomiting, minor mucosal bleeding, pleural effusion, ascites and hepatomegaly. Patients with shock, respiratory distress, severe bleeding and/or organ impairment were classified as severe dengue. Healthy controls were matched to age, sex and socio-economic status and recruited in the same geographical area as the study subjects.

### Laboratory diagnostics

#### Serology

All diagnostic assays were performed at the Department of Microbiology, Faculty of Medicine, University of Indonesia, Jakarta. The diagnosis of the rapid test was confirmed with detection of NS1 antigen (Panbio) and/or IgM antibodies (Focus diagnostics) and/or a positive RT-PCR (see next section). IgM and IgG antibodies were determined in all collected sequential samples. Patients with a positive IgG in the sample from day 3–4 after admission were considered as secondary DENV infection, while patients with a negative IgG in this sample were considered as a primary DENV infection. In two patients viral RNA could not be detected, but they were included, because these patients were NS1 and IgM positive.

#### Molecular diagnostics

Reverse transcriptase PCR (RT-PCR) was performed to determine the infecting serotype according to the method described in Lanciotti et al [10]. RNA was extracted from 140 μl of plasma using QIAamp viral RNA mini kit (Qiagen, Germany) according to the manufacturer’s instruction. RNA isolation and PCR were performed in strict containment to avoid contamination. Negative controls were included in both RNA isolation and in RT-PCR.

A semi-nested RT-PCR was performed for serotyping. The first amplification reaction had a reaction volume of 40 μl, consisting of 4 μl 10x PCR buffer with 1.5 mM MgCl_2_, 3.2 μl 10mM dNTPs, 0.4 μl Super Script II RTase (Invitrogen), 0.15μl 5 U Platinum Taq-polymerase (Invitrogen), 0.8 μl 10 μM D1 primer, 0.8 μl 10 μM D2 primer, 8 μl RNA. The RT step consisted of 53°C for 30 minutes and denaturation at 95°C for 5 minutes. This was followed by 30 cycles of denaturation at 95°C for 45 seconds, annealing at 60°C for 30 seconds, and extension at 72°C for 90 seconds. The RT-PCR was concluded with a single cycle extension at 72°C for 7 minutes.

The second PCR amplification had a reaction volume of 25μl, consisting of 2.5μl 10x PCR buffer with 1.5 mM MgCl_2_, 2.0 μl 10 mM dNTPs, 0.15μl 5 U/μl Platinum Taq-polymerase (Invitrogen), 1μl of each D1, TS1, TS2, TS3, TS4 primer (10 μM), 2μl of the product from the first PCR. PCR 2 was performed with initial denaturation at 95°C for 5 minutes, followed by 35 cycles of denaturation at 95°C for 45 seconds, annealing at 60°C for 30 seconds, extension at 72°C for 60 seconds and one final single cycle extension at 72°C for 7 minutes.

After amplification, PCR products were analysed by electrophoresis. Eight μl of PCR product was loaded on 2% agarose gel in TAE buffer and stained with Ethidium Bromide. The product was visualized with UV light.

#### Blood transcriptome profiling

Blood was collected in Tempus Blood RNA tubes (ABI, Foster city, CA, USA). Total RNA was isolated from whole blood using the Tempus Spin RNA isolation kit (Applied Biosystems, Bleiswijk, The Netherlands). Globin RNA was removed from total RNA preparations using the Globiclear kit (Life Technologies). RNA concentrations and OD 260/280 ratios were measured with the NanoDrop ND-1000 UV-VIS spectrophotometer (NanoDrop Technologies, Wilmington,USA). Assessment of RNA quality and purity was performed with the RNA 6000 Nano assay on the Agilent 2100 Bioanalyzer (Agilent Technologies, Palo Alto, CA, USA). RNA (100 ng) was labelled using the MessageAmp Premier RNA Amplication kit (Applied Biosystems) and hybridized to Human Genome U133 plus 2 gene chips (Affymetrix), according to the manufacturer’s recommendations. Image analysis was performed using GeneChip Operating Software (Affymetrix). Microarray Suite version 5.0 software (Affymetrix) was used to generate .dat and .cel files for each experiment. The raw data has been deposited in the arrayExpress database under access number E-MTAB-3162.

#### Data normalization and analysis

Analysis of the arrays was performed using R 2.15/Bioconductor [11,12]. Gene expression was evaluated using alternative probeset definitions based on the ensembl genome probeset annotation that yield a single probeset per gene. After quality control and VSN normalization [13–15], probeset summarization was performed by median polish (i.e., RMA). Differential gene expression and Reactome pathway analysis was performed using Limma and the Roast routine [11,16]. Limma fits a linear model for every gene and tests for differential expression. The t-statistics are then moderated using an empirical Bayes-approach. Roast uses a similar approach to perform a self-contained gene set test for gene sets. Significance for gene sets is computed by a bootstrapping approach (n = 10000). Multiple testing correction (FDR < 0.05) is applied to both differential gene lists as well as differential pathway lists.

#### Network construction

We applied weighted gene correlation network analysis (WGCNA) using the available R libraries [17]. This method constructs a continuous correlation network with a scale-free network topology. Our pre-processing approach yielded single probeset-gene pairs with no ‘absent calls’ for any gene (see previous Methods section) that not require any additional filtering. This allowed us to use all available probesets for network construction and ensured that every gene was only included in the network once as a single node, thereby providing an optimal approximation of the dengue transcriptional network structure. This co-expression network was subsequently divided into modules using a topological overlap distance measure and the dynamic tree cut algorithm. Gene modules are summarized by the first principle component of the module, which can be correlated to parameters associated with the cohort. External datasets were incorporated by using the list of differentially expressed genes made available by the authors in the original paper.

## Results

### A longitudinal cohort of dengue patients

During the 2010 dengue outbreak in Jakarta, Indonesia, 157 patients were recruited into this study. Of these patients, 52 were admitted to the hospital with a positive dengue IgM and/or NS1 rapid test. All four dengue serotypes were circulating during this outbreak. Out of the 52 admitted patients, 26 patients were selected for blood transcriptome analysis based on the completeness of clinical data (i.e. symptoms, ultrasound data, laboratory parameters), and the availability of samples from day 0 and day 4 of admission and a confirmed laboratory diagnosis of the rapid test for DENV infection (see Table 1 for clinical characteristics). During hospital admission, seven patients were diagnosed with WS-, eighteen with WS+ and one with severe dengue. The patient with severe dengue displayed signs of severe haemorrhage, including melaena. No patients developed shock, although fifteen patients did receive a large amount of IV fluid during their admission (more than 10 litres in total and one patient even 25 liters), indicating that these patients were critically ill. Interestingly, the leukocyte count was not different between day 0 of admission (median 4070, (IQR: 2575–5380)) and day 4 (median: 3930, (IQR: 2495–5355)), although at day 0 the leukocyte count was lower in the WS+ group compared to the WS- group, but this difference was not significant (Table 1). Fifteen age- and sex-matched healthy controls from the same geographical location in Jakarta and similar socio-economic status were also included in this study.

**Table 1:** Clinical characteristics of the cohort.

2009 WHO dengue case classification	WS- (N = 7)	WS+/severe (N = 19)
Sex (male/female)	3/4	11/8
Age*	20 (17–33)	19 (17–27)
Primary/secondary/unknown	2/4/1	6/13/0
Serotype (DENV-1/-2/-3/-4/not detected)	3/2/1/0/1	2/6/7/3/1
Pleural effusion during admission (US)	0	4 (21%)
Ascites during admission (US)	0	9 (47%)
IV fluid during admission (ml)*	7000 (2000–11000)	11000 (6000–13500)
Positive tourniquet at day of admission	2 (33%)#	10 (53%)
Leukocyte count at admission*	6060# (2655–7470)	4000 (2520–5150)
Thrombocytopenia during admission	3 (50%)#	18 (95%)
Mucosal bleeding	0	6 (32%) (1 gum, 2 epistaxis, 2 gum and epistaxis, 1 melaena)
Vomiting during admission	0	6 (32%)
Abdominal pain during admission	0	12 (63%)

* Values are in median (interquartile range). Abbreviations: WS-: non-severe dengue without warning signs. WS+: non-severe dengue with warning signs. # = 1 missing value in the WS- group in the tourniquet test, platelet and leukocyte count.

A maximum of three tempus tubes from each patient was included in this analysis. We included a total of 61 tempus tubes from 26 patients. 20 tempus tubes were collected at day 0 of admission (i.e., day 1–2 after onset of fever) and 20 at day 4 (i.e., day 4–5 after onset of fever); these time points were therefore analysed in detail. An overview of the timepoints and disease categories is provided in S1 Table.

### Time since admission accounts for most transcriptome variance

**Fig 1:**
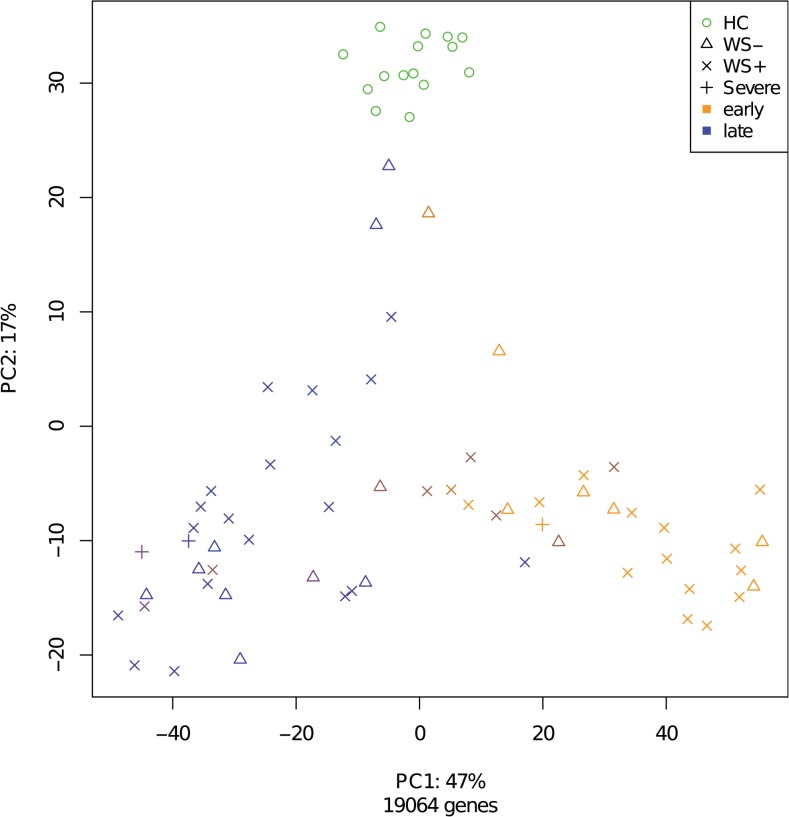
Principle component analysis of all transcriptome snapshots in this study. Icons and colours indicate type and sampling stage of the samples. Timing of samples range from day 0 to day 6 of admission. The day 0 samples have the lightest colour and the day 6 samples the darkest. All probesets were included for this analysis.

To obtain a global overview of the dengue transcriptome profiles, we applied principal component analysis (PCA) (Fig. 1). This non-supervised analysis method finds the ‘optimal point of view’ for observing differences between the samples and depicts this as a distance in a 2-dimensional plot. The first principle component (PC1) accounts for 47% of the variance in the dataset and concurs with time since admission. The second principle component (PC2) accounts for 17% of the variance in gene expression and segregated the dengue samples from the healthy controls; together, PC1 and PC2 account for 64% of gene expression differences in the dataset. PCA did not show any segregation of patients by disease severity according to the 2009 WHO classification. Taken together, PCA demonstrates that time since admission has the highest impact on the dengue transcriptome profiles in our cohort.

### WS- and WS+ dengue patients have indistinguishable transcriptional profiles

**Fig 2:**
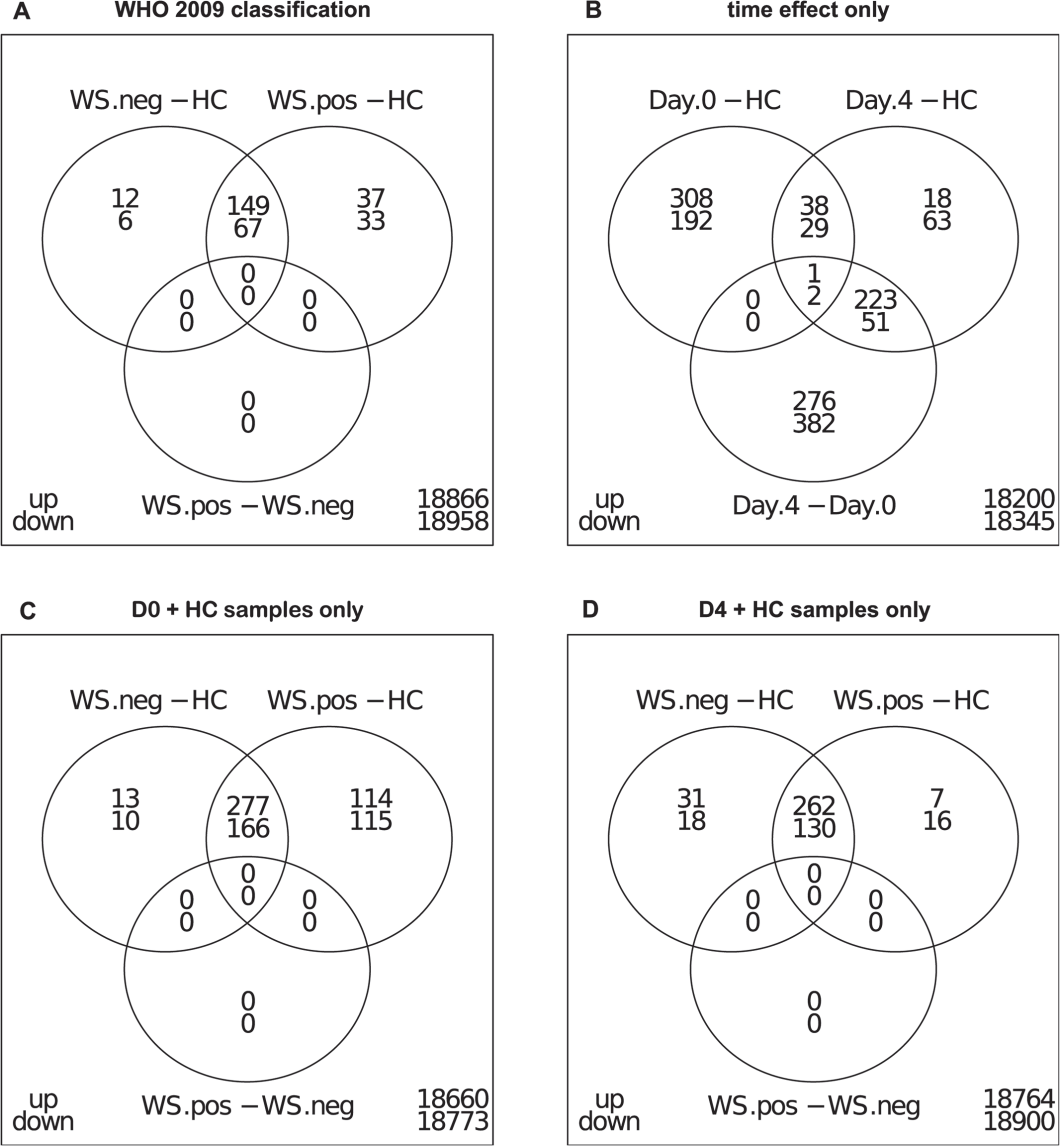
Differential gene expression analysis. Venn diagrams show the overlap between different sets of differentially expressed genes. FDR <0.05, all differentially expressed genes are at least 2-fold up- or down-regulated.

To obtain insight into the transcriptional changes that are associated with disease severity and time since admission, we performed differential gene expression analysis and gene set analysis in dengue patient and control transcriptomes (FDR ≤ 0.05 and fold change ≥ 2). We used the 2009 WHO dengue case classification system to group patients and excluded the single case with severe disease from gene expression analysis. Combining data from all time points revealed that 161 genes were up- and 73 genes were downregulated in WS- patients compared to healthy controls (Fig. 2A, S1 Information). In WS+ dengue patients, 186 genes were up- and 100 genes were downregulated relative to healthy controls. There is considerable overlap (216 genes) of differentially expressed genes in both dengue groups, suggesting that similar biological processes are ongoing in both WS- and WS+ dengue patients. Indeed, no genes were differentially expressed when comparing WS- to WS+ dengue patients directly. Next, the transcriptome profiles of samples from day 0 and day 4 since admission were compared to identify genes differentially expressed over time, regardless of disease severity (Fig. 2B, S1 Information). More genes were differentially expressed in time since admission than between WS- and WS+ disease, confirming the PCA results that time since admission has the largest impact on the transcriptome. To study dengue disease effects independently of time since admission, we restricted our analysis to transcriptome profiles from WS-, WS+ and healthy controls at day 0 and day 4 of admission. On day 0, many genes were differentially expressed in each of both dengue groups compared to healthy controls, but no genes were differentially expressed when the severity groups were compared to each other (Fig. 2C). At day 4, the number of differentially expressed genes in WS- and WS+ dengue compared to healthy controls was lower than at day 0 (Fig. 2D, S1 Information). When severity groups were compared at day 4, again no genes were differentially expressed. Taken together, in our study, WS- and WS+ blood transcriptional profiles cannot be distinguished from each other.

### Time since onset of disease explains discordant transcriptional signatures in dengue patients

**Fig 3:**
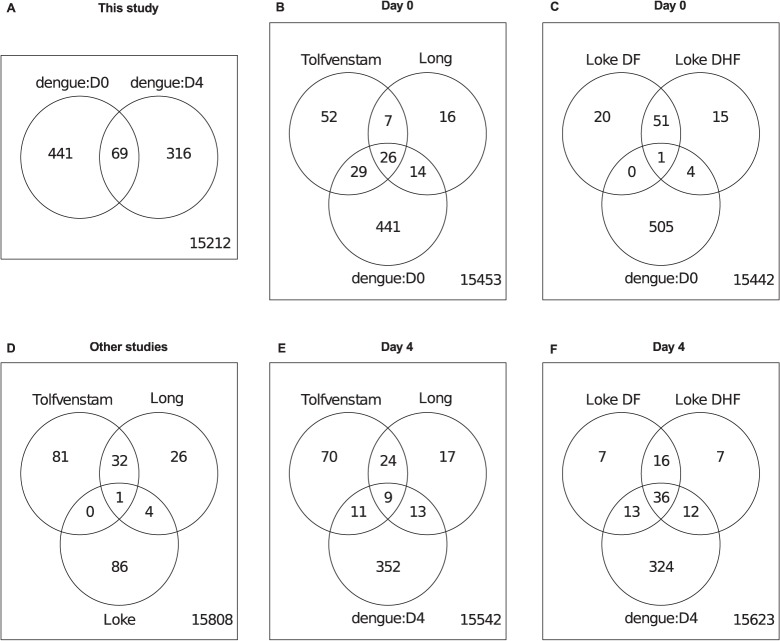
Overlap of differential gene expression with signatures identified in other studies. (a) indicates the signatures generated in this study, (d) those of the other dengue studies. (b-c) and (e-f) indicate the overlap with the signatures of day 0 and day 4, respectively.

Over the past few years, several studies have examined the transcriptional profile of dengue infections. Three cross-sectional studies (Tolfvenstam et al., Long et al. and Loke et al.) [6,7,18] were similar in the type of sample used (whole blood) and the included data on time since onset of symptoms, allowing these studies to be compared to results from our cohort (Fig. 3A, Table 2). Tolfvenstam et al. and Long et al. have a fairly large overlap in differentially expressed genes (Fig. 3D), presumably because both studies included patients early (<72 hours) after onset of disease. The signatures published by Loke et al. have little overlap with those of the other studies (1 and 5 genes only, Loke et al. DF and DHF signatures combined), most likely due to the fact that patients were included at a later time point after the onset of disease (3–6 days after onset). To compare our results to these studies, we compared the early and late general dengue signatures to those of the other studies. 48% of differentially expressed genes in the signature from Tolfvenstam et al. and 63% of Long et al. are also part of our day 0 dengue gene signature (collected <48 hours after onset of disease) (Fig. 3B). On the contrary, only 1% of the genes in the DF and 7% of the genes in the DHF signature in Loke et al. were similar to our day 0 signature (Fig. 3C). In contrast, our day 4 signature showed the greatest similarity with the signatures in Loke et al. (68% DF, 68% DHF; Fig. 3F), but much less so with those from Tolfvenstam et al. and Long et al. (18% and 35%, respectively; Fig. 3E). Our results therefore concur with all three studies and confirm that these signatures can occur within one cohort, but at different time points after onset of symptoms. In conclusion, time since the onset of symptoms accounts for most of the transcriptome differences between mRNA profiling studies in dengue patients.

**Table 2:** Characteristics of studies profiling whole blood of DENV infected patients.

Study	Loke et al.	Long et al.	Tolfvenstam et al.
Journal	PloS Neglected Tropical Diseases	Journal of Virology	BMC Infectious Diseases
Year	2010	2010	2011
Country	Nicaragua	Vietnam	Singapore
Population	Children < 15 years	Adults and children	Adults
Day of illness	Between 3–6 days	<72 hours	<72 hours

### The dengue transcriptome shifts from an immunity and inflammation—to a repair and recovery signature

**Fig 4:**
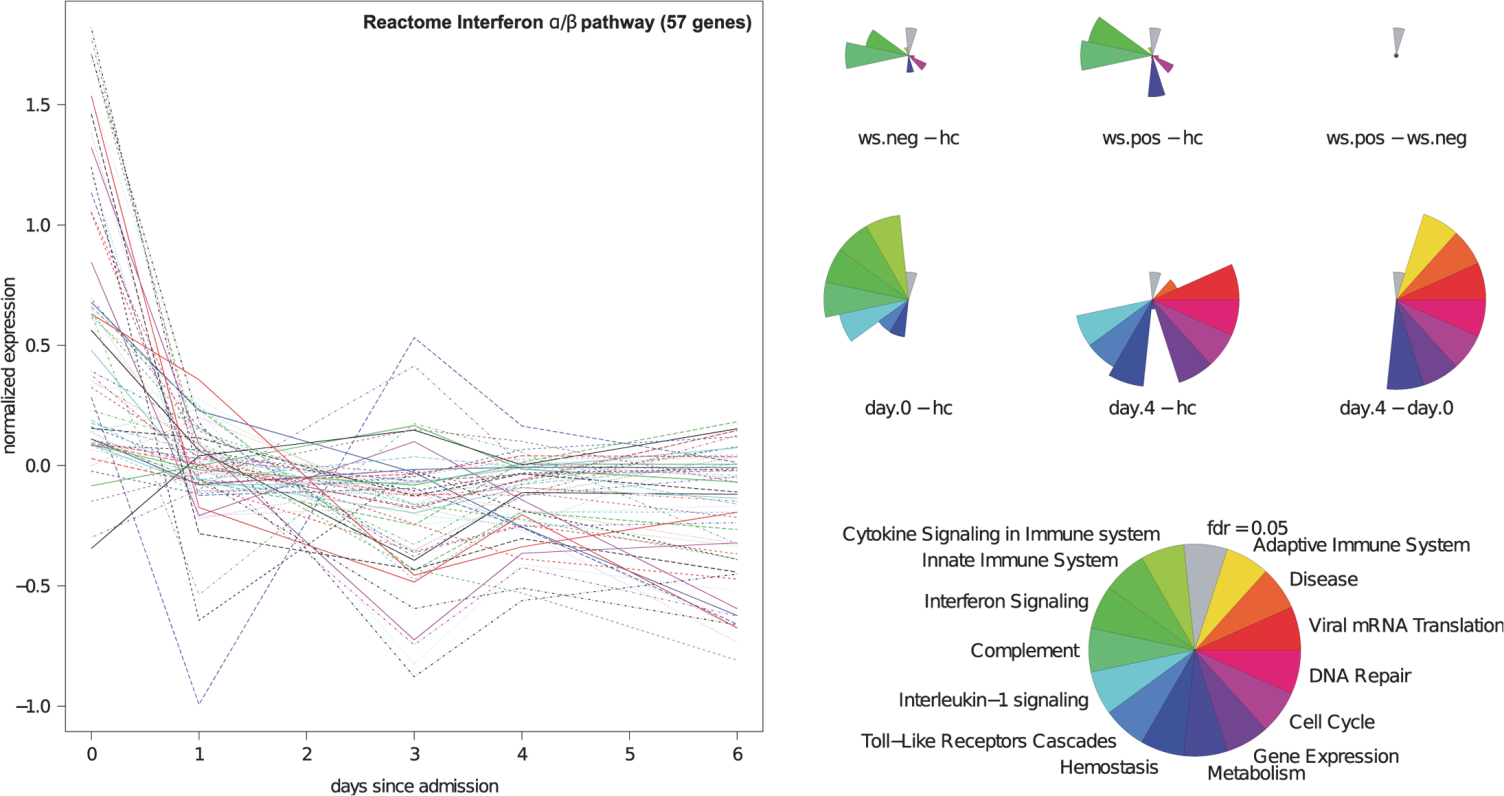
Gene set analysis of dengue signatures. The interferon pathway (left panel) is upregulated in the early stages of dengue, but not later. Segment plots indicate the significance of enrichment of particular Reactome gene sets and pathways. The longer the segment, the larger the enrichment. All segments longer than the grey segment are significant.

The type I interferon pathway is known to be differentially expressed in DENV infection [19]. We investigated the interferon response during the course of infection by selecting genes from the interferon pathway (Reactome curated pathway database, 57 genes) and plotting their expression over time (Fig. 4). The expression of the majority of interferon genes was highly increased on the first day of admission, but decreased rapidly after that day and continued to be low, consistent with the notion that the interferon pathway is active in the early stages of dengue infection [4].

To functionally annotate the differences in gene expression, we performed gene set analysis using the Reactome curated pathway database [20]. Applying the Roast algorithm [21], we found that when compared with healthy controls, both WS- and WS+ have ‘complement’ and ‘interferon signalling’ ranked among the most highly up-regulated pathways (Fig. 4, S2 Information). None of the pathways were differentially regulated when comparing the WS- with the WS+ patients.

In addition to severity signatures, we investigated the transcriptional changes related to time since admission in our longitudinal cohort. By comparing day 0 dengue samples to healthy control samples, we observed an up-regulation of pathways related to innate immunity and cytokine signalling (Fig. 4, S2 Information). When we compared Day 4 to healthy controls, a pronounced shift to cell cycle and DNA repair mechanisms was evident. A direct comparison between day 0 and day 4 samples additionally showed up-regulation of metabolism (Fig. 4). Taken together, we see that, initially, innate immunity and interferon is up-regulated, followed by repair mechanisms that mark the beginning of recovery from dengue.

### Classical dengue clinical parameters associated with transcriptional patterns in dengue

**Fig 5:**
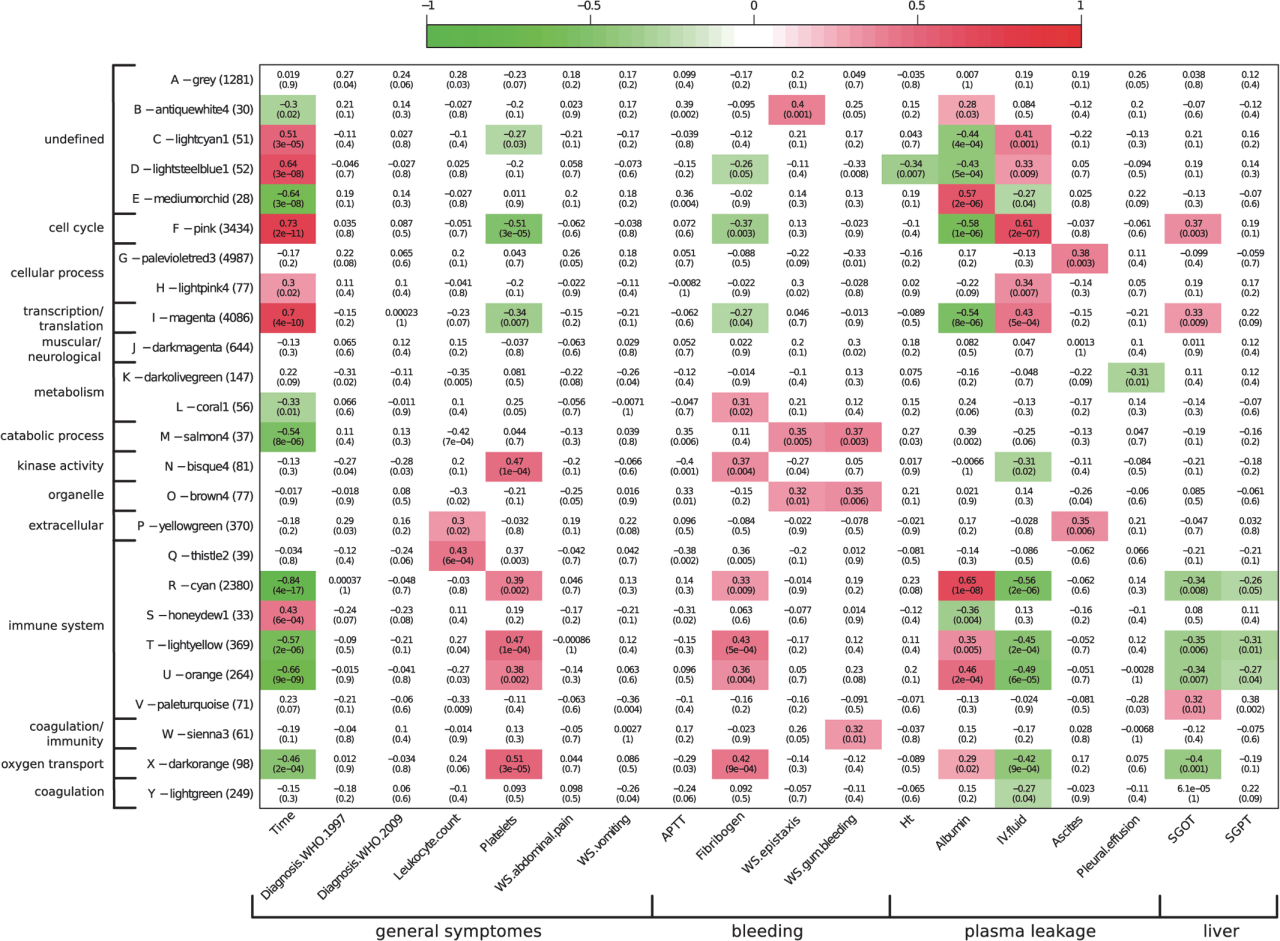
Dengue co-expression network analysis. Significant correlations between gene modules (y-axis) and clinical parameters (x-axis) are depicted in a red-to-green colour scale. Upper number is pearson correlation coefficient, lower number the level of significance (p-value). All gene modules are annotated with gene enrichment categories; clinical parameters are grouped by symptom type.

Next, we investigated the association between the identified gene modules and clinical parameters. To this end, we used weighted gene correlation network analysis (WGCNA) that organizes genes into 25 modules that are subsequently correlated to 18 clinical parameters (Fig. 5, S3 Information). This analysis confirms that time since admission has a strong effect on the transcriptome of dengue patients and that immune-related genes dominate the early response. Significant associations between gene modules and the clinical parameters platelet count, fibrinogen level, albumin level and volume of IV fluid per day were found. Most modules that displayed a positive correlation with time after admission also did so with the quantity of IV fluid and the liver enzyme SGOT. These same modules displayed a negative association with the platelet count and levels of fibrinogen and albumin. Platelets, albumin and fibrinogen are all part of the blood compartment in which dengue targets monocytic cells to replicate [22]. The pro-inflammatory environment due to DENV replication probably affects the expression of these markers. This may explain the association of these markers with these gene modules.

In contrast to the above-mentioned clinical parameters, the 1997 WHO dengue case classification as well as the 2009 WHO dengue case classification showed no significant association with any of the gene modules, suggesting that these classifications do not reflect the underlying biological processes, or that there are no differences in the underlying biological processes. The non-specific warning signs ‘abdominal pain’ and ‘vomiting’ showed no statistical significant associations, which is in line with the generic nature of these symptoms. In contrast, DENV-specific warning signs including ‘epistaxis’ and ‘gum bleeding’ did correlate with the gene modules B-antiquewhite4, O-brown4, M-salmon4 and W-sienna3. Enriched GO terms for genes in the O-brown4 module are “cell organelles”, such as mitochondrion and cytoplasm. Module W-sienna3 has wound healing and coagulation as enriched GO terms and module M-salmon4 is related to catabolic processes. Ascites, which is a typical sign of plasma leakage, is significantly associated with modules G-palevioletred3 and P-yellowgreen. These two gene modules were not associated with time, suggesting that this is a specific biological pathway. All in all, network analysis showed that the WHO classifications couldn’t be related to specific gene modules; however, there are many significant correlations between gene modules and dengue-specific clinical parameters.

### Specific genes correlate with the platelet count and the liver enzyme SGOT

**Fig 6:**
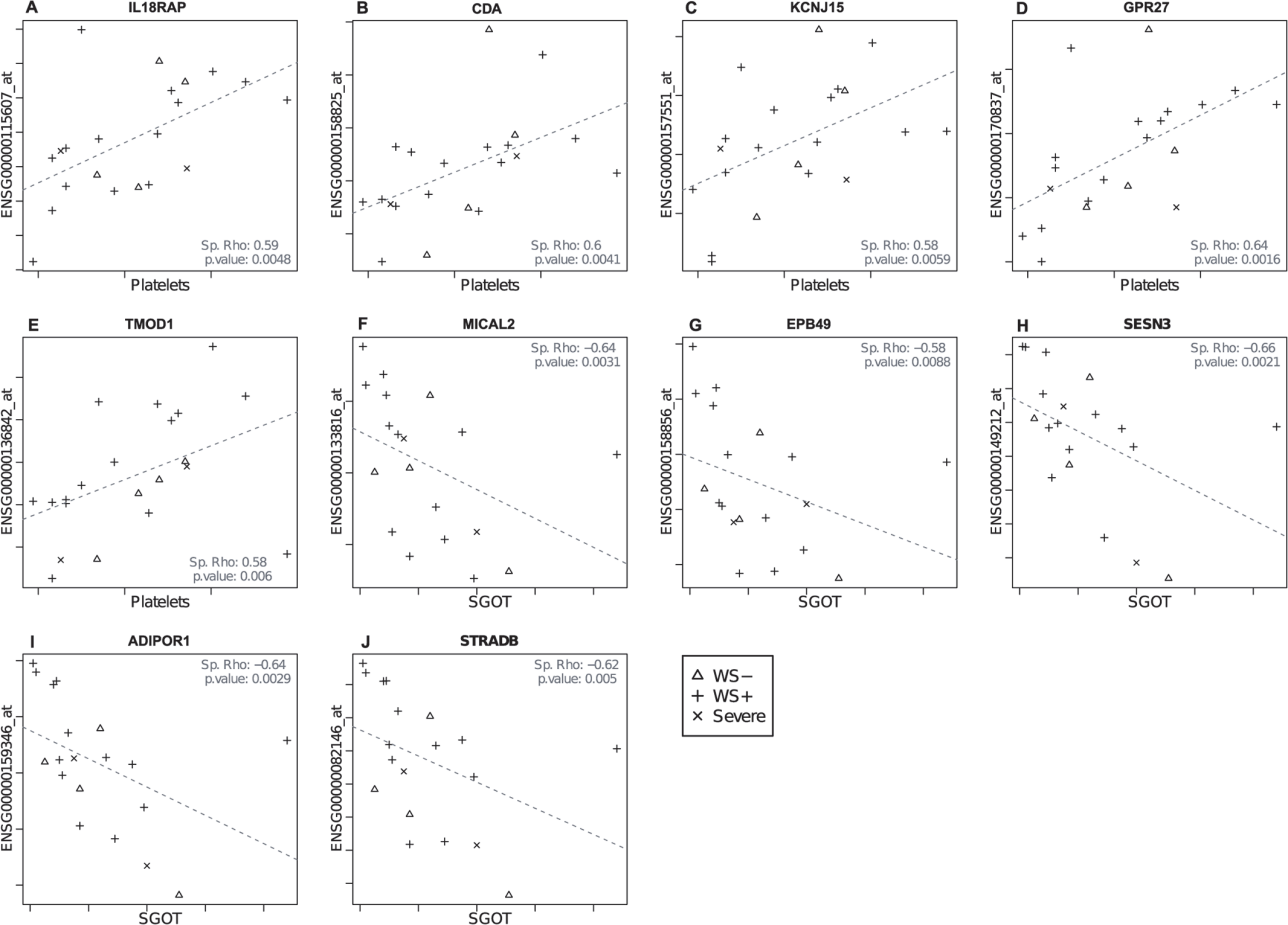
Correlations between genes (y axis) and clinical parameters (x-axis). Genes are annotated with a gene symbol; those depicted in (a-d) are from module T-lightyellow; (e-j) are from module X-darkorange. Icons depict the class of the sample (see Fig. 1). Spearman’s Rho correlations and p-values are given in the plot.

In order to identify genes that may serve as a marker of immune activation in the early phase of disease, we focused on modules with a strong negative correlation with time after admission. These modules contain genes that are upregulated specifically in the earliest phase of disease that could represent biomarkers for disease progression, including modules M-salmon4, R-cyan, T-lightyellow, U-orange, and X-darkorange. By correlating genes contained in these modules with clinical parameters that mark disease severity, genes associated with can be identified. Five genes in the module T-lightyellow showed a significant direct association with the platelet count (Fig. 6), including two that play a role in immunological processes. The IL-18 receptor accessory protein (IL-18RAP, Fig. 6A) forms the receptor complex with IL-18Rα and is needed for IL-18 signalling. Cytidine deaminase (CDD, gene CDA) is highly expressed by activated granulocytes and serves as a negative feedback mechanism of these cells by inhibiting the function of granulocyte-macrophage colony formation in the bone marrow [23] (Fig. 6B). KCNJ15 and G-protein coupled receptor 27 (GPR27) (Fig. 6C-D) are both described to play a role in insulin secretion [24,25]. In the module X-darkorange, the gene Tropomodulin 1 (TMOD1) was directly associated with the platelet count (Fig. 6E) and the genes Mical2 and dematin (gene EBP49) with SGOT (Fig. 6F-G) all play a role in actin regulation of the cell [26–28]. Seven other genes with an inverse association with SGOT play a role in metabolic processes, including sestrin 3 (SESN3), adiponectin receptor 1 (ADIPOR1) and STE20-related kinase adaptor beta (STRADB) (Fig. 6H-J). Sestrin 3 is required for regulation of the blood glucose levels [29] and sestrins can reduce the levels of reactive oxygen and protect cells against cell death [30]. Adiponectin acts through the adiponectin receptor 1, which results in increased fatty acid oxidation in the liver [31]. Adiponectin activates serine/threonine kinase 1 via its receptor (LKB1) [31]. Interestingly, the gene STE20-related kinase adaptor beta was also significantly associated with the SGOT and is part of a complex involved in the activation of LKB-1. LKB-1 is important in maintaining cell polarity of hepatocytes, which is essential in the formation and maintenance of the bile canalicular network [32]. In summary, we find genes that correlate with clinical parameters and that can be related to either dengue pathogenesis or tissue physiology, suggesting that these genes may be directly associated with the ongoing biological processes in dengue infection.

## Discussion

In this study, we examined DENV infected patients with uncomplicated disease using transcriptome profiling. By using a variety of analysis techniques, we show that time since admission, which is a proxy for time since onset of disease, is the most important determinant of the blood transcriptome profile changes in DENV infected patients. Regardless of the analysis techniques used, we did not observe differences in blood transcriptional profiles between WS- and WS+ patients. Conversely, the clinical parameters platelet count, albumin, fibrinogen, SGOT, SGPT and volume of IV fluid administered showed a highly significant association with specific gene modules. Gene module expression may serve as a novel marker to monitor the biological processes involved in dengue pathogenesis.

A recurring theme in our results is that time after the onset of disease is the main determinant of transcriptome profile changes in DENV infected patients. This observation is in line with the results reported by Sun et al., although PBMCs were used in that study [5]. Moreover, the comparison of our data to signatures published by Loke et al., Long et al. and Tolfvenstam et al. show that stratifying the expression data by time after onset of disease results in a large overlap with these gene expression profiles. These studies performed transcriptome profiling in unrelated cohorts from populations of patients with diverse genetic backgrounds, geographical locations and age distributions, demonstrating that the impact of time since onset of disease on gene expression is a general and robust feature of DENV infection.

In contrast to the strong signal related to time since onset of disease, there was no detectable transcriptional difference between WS- and WS+ patients. Earlier studies showed that gene expression patterns from patients with DSS did segregate from those of DF and DHF patients [9,18]. This is the first time that the 2009 WHO classification was used in transcriptome analysis, but our results extend other studies showing that no clear distinction can be made between the transcriptome profile of DF and DHF samples [9,18,33]. This conclusion is based on differential gene expression and co-expression network analysis of dengue transcriptome data that both take all available genes into account; it is therefore unlikely that this is due to a gene inclusion bias during the analysis phase. The fact that no differentially expressed genes could be identified in comparisons between WS- and WS+ dengue suggests that the biological processes in these two disease entities are very similar or that the generated blood transcriptome profiles do not accurately reflect disease processes in other parts of the body. The latter is not expected given that DENV infection is a systemic disease that targets monocytic cells in the blood compartment [22]. Our network analysis showed that epistaxis, gum bleeding and ascites were associated with gene modules distinct from those that associate with markers reflecting systemic disease, such as platelet count, fibrinogen, albumin and IV-fluid, suggesting that these markers reflect different biological processes. The network analysis also showed that the parameters platelet count, fibrinogen, albumin and IV fluid reflect the processes of systemic immune activation and subsequent repair mechanisms in the blood. We conclude that distinct dengue-related signatures can be identified, but that these do not concur with the comprehensive WS- and WS+ categories in dengue diagnosis. Furthermore, if WS-/WS+ specific biomarkers could be identified, the strong effect of time upon infection on transcriptome dynamics would limit the application of such biomarkers in a clinical setting. However, the expression level of the identified gene modules specific for biological processes relevant in dengue disease may support earlier detection of progress to severe disease and improve clinical management of dengue.

The clinical parameter platelet count has frequently been associated with DENV infection [34] and was even one of the four criteria for DHF in the 1997 WHO dengue case classification [35]. It has been shown that children with lower platelet counts in the early phase of disease are more likely to develop DHF later on [36]. Several hypotheses to link DENV infection with platelet depletion have been postulated, such as DENV induced bone marrow suppression [37], complement-induced lysis of platelets through the binding of autoantibodies [38] or the binding of platelets to activated endothelial cells [39]. Our study finds, besides an association between the platelet count and certain gene modules, a strong activation of the innate immunity and complement, which could contribute to all these three mechanisms of platelet depletion. Furthermore, we found that expression of IL-18RAP, which is involved in the induction of IFN-γ production in NK and Th1 cells [40], to be directly associated with the platelet count. Fagundes et al. showed that IL-18 signalling was necessary to inhibit viral replication in DENV infected mice and that IL-18 knock-out mice showed increased virus titres and more severe disease [41].

The occurrence of plasma leakage in dengue patients has been extensively documented. We observe that markers of plasma leakage, including the quantity of IV-fluid supplied and the levels of albumin, were both associated with the same gene modules as platelet count. Plasma leakage tends to correlate inversely with the platelet count [42] and it has been suggested that platelets may directly induce vascular permeability by the release of IL-1β [43]. Albumin is strongly negatively charged, which prevents leakage from the circulation under normal conditions. However, decreased levels of albumin have been detected in patients with DSS, suggesting that selective restriction by the endothelial barrier is impaired during DENV infection, resulting in leakage of albumin from the circulation to the tissue [44]. In different cohorts of patients, we showed that dengue shock syndrome and a pro-inflammatory cytokine profile were strongly associated with microbial translocation and the presence of lipopolysaccharide (LPS) in the blood, suggesting that plasma leakage is the result of immune activation during DENV infection [45]. In this study, we find that the R-cyan module is strongly associated with albumin concentration and plasma leakage; the module’s contents show an association with pro-inflammatory cytokines of the interferon response, as well as upregulation of the TLR-4 pathway that detects LPS. This result is in line with our previous observations [45,46], that link pro-inflammatory cytokines profiles, microbial translocation and plasma leakage in DENV infection.

In severe dengue, dysregulation of coagulation is frequently observed. Fibrinogen is consumed after thrombin generation and decreased levels have been detected in severe dengue [47]. The strong association of fibrinogen with gene modules involved in immunity and inflammation suggests that activation of the coagulation cascade is associated with the strong immune response in the acute phase of DENV infection. It has been shown that activation of the coagulation cascade can induce the production of cytokines through NF-κB activation [48], indicating that crosstalk between coagulation and inflammation may contribute significantly to disease severity.

In our study, gene modules were enriched for metabolic and catabolic processes, kinase activity and organelles, such as mitochondria. Loke et al., also showed that many upregulated genes in the acute phase were involved in metabolic processes and shared between acute DF, DHF and DSS samples [18], suggesting that a highly activated metabolic state is part of the general dengue signature. Acute DENV infection is also characterized by extensive activation of the innate immune response, such as complement and neutrophils [5,7,49]. Complement is suggested to be an important inhibitor of replication of Flaviviruses [50], and was among the most highly overrepresented pathways in our study. Similarly, studies performed in whole blood have shown that neutrophil-related genes are highly expressed in dengue expression signatures that correlated with disease severity [3,9,18]. In our study, the neutrophil derived protein CDD associated with the platelet count, suggesting increased abundance in the acute phase of disease. This protein could be associated with severe disease, because high levels of CDD have also been shown in patients with meningococcal sepsis [51].

Our results indicate involvement of the liver in dengue pathogenesis. Especially the gene modules involved in inflammation and immunity correlated with the liver enzymes SGOT and SGPT, suggesting that dengue induced inflammation affects the liver. Moreover, ten individual genes showed a significant association with the liver enzyme SGOT. The liver may be affected directly by viral replication or indirectly by cytokines and immune cells. One surprising finding from our study is differential regulation of several genes that play a role in diabetes. The genes adiponectin receptor 1, sestrin 3, KCNJ15 and G-protein coupled receptor 27 have all been described to play a role in insulin resistance, decreased insulin secretion and impaired blood glucose homeostasis [24,25,52]. It has been shown that inflammation and certain cytokines in particular lead to insulin resistance, which may result in further activation of multiple inflammatory processes [53,54]. In support of this finding, it has been shown that DENV infected patient with diabetes had a higher risk to develop severe disease [55,56]. Altogether, the above suggests involvement of the liver in dengue pathogenesis, in particular related to insulin and blood glucose regulation during DENV infection and pathogenesis.

Future studies should aim to carefully track time since the start of infection, as time is the main source of variance in transcriptomes from dengue patients. Since the transcriptome effects in time are larger than potential transcriptome effects between severity classes, a synchronous longitudinal cohort is an absolute requirement for any biomarker study. Furthermore, individual symptoms and markers appear to better reflect the biological processes underlying dengue pathogenesis. Classification on the basis of gene signatures related to specific symptoms, rather than overall diagnosis, may enable earlier identification of patient subgroups that are at increased risk of developing severe dengue, and to a better understanding of dengue disease pathogenesis.

## Supporting Information

S1 Checklist STROBE checklist. (DOC)

S1 Information Results for differential gene expression analysis as assessed by Limma analysis (false discovery rate > 0.05, fold change ≥ 2). For every contrast, the probeset ensemble genome identifier, the average expression, the log fold change in expression, and the adjusted p value (i.e., fdr) are given.
(XLSX)

S2 Information Results of gene set analysis as assessed by Roast analysis (false discovery rate > 0.05). For every contrast, the proportion active genes and significance (false discovery rate) are given for every pathway. In every case, pathway differential regulation is assessed for up-regulated genes, down-regulated genes, and for up- or down-regulated (‘mixed’) genes.
(XLSX)

S3 Information Results from the WGCNA analysis that confirms some of the findings of the differential gene expression analysis. (DOCX)

S1 Table Characteristics of samples included in transcriptome profiling in this study. WS- and WS+ indicate non-severe dengue without and with warning signs, respectively.
(DOCX)
