# Supplementary material for: Time since Onset of Disease and Individual Clinical Markers Associate with Transcriptional Changes in Uncomplicated Dengue
Source: PLoS Negl Trop Dis. 2015 Mar 13;9(3):e0003522. doi: 10.1371/journal.pntd.0003522 (PMC4358925; doi:10.1371/journal.pntd.0003522)
Supplement: S3 Information — (DOCX) [file pntd.0003522.s004.docx]

**Supplementary Results for**

**Time since Onset of Disease and Individual Clinical Markers Associate with Transcriptional Changes in Uncomplicated Dengue**

Cornelia AM van de Weg^1#^, Henk-Jan van den Ham^1#^, Maarten A Bijl^1^, Fatih Anfasa^1,2^, Fatiha Zaaraoui-Boutahar^1^, Beti E Dewi^3^, Leonard Nainggolan^2^, Wilfred FJ van IJcken^4^, Albert DME Osterhaus^1^, Byron EE Martina^1^, Eric CM van Gorp^1^, Arno C Andeweg^1^.

# These authors contributed equally to this study

^1^Department of Viroscience, Erasmus Medical Center, Rotterdam, The Netherlands

^2^Department of Internal Medicine, Faculty of Medicine, University of Indonesia, Jakarta, Indonesia

^3^Department of Microbiology, Faculty of Medicine, University of Indonesia, Jakarta, Indonesia

^4^ Department of Biomics, Erasmus Medical Center, Rotterdam, The Netherlands

*Network analysis confirms early immune activation*

In addition to conventional differential gene expression analysis, we applied weighted gene correlation network analysis (WGCNA) to identify novel biological processes that are involved in DENV infection and pathogenesis. WGCNA organizes genes into modules that are subsequently correlated to clinical parameters. Using this approach, we identified 25 gene modules (including one group of unassigned genes) of varying sizes and annotated them using Gene Ontology [[55](#_ENREF_55)]. All modules were labelled with a functional category that is enriched in this module (Figure 5, left column). Furthermore, the gene expression patterns of the modules were compared with 18 clinical parameters (Figure 5, red and green indicate a positive or negative correlation, respectively). Two modules did not associate with any clinical parameter: Module J-darkmagenta consisted almost exclusively of inactive genes; A-grey is a group of genes that do not cluster – and it is therefore not surprising that these clusters do not correlate with any clinical parameter. Time since admission correlated strongly with a large number of gene modules, confirming that time has a strong effect on the transcriptome of dengue patients. Furthermore, most gene modules involved in immunity showed a negative correlation with time since admission, while the gene modules involved in cellular processes and cell repair mechanisms showed a positive correlation with time. This demonstrates that gene expression in the early phase of disease is dominated by immune response genes, which shifts to genes involved in cell repair mechanisms around the time of defervescence. The modules R-cyan and T-lightyellow had a strong inverse correlation with time. The R-cyan module is enriched for genes involved in the type I interferon response, innate immune response, cytokine production and toll-like receptor 4 signalling pathway; the T-lightyellow module for inflammatory response and lymphocyte and neutrophil activation.
