# Supplementary material for: Time since Onset of Disease and Individual Clinical Markers Associate with Transcriptional Changes in Uncomplicated Dengue
Source: PLoS Negl Trop Dis. 2015 Mar 13;9(3):e0003522. doi: 10.1371/journal.pntd.0003522 (PMC4358925; doi:10.1371/journal.pntd.0003522)
Supplement: S1 Table — WS- and WS+ indicate non-severe dengue without and with warning signs, respectively. (DOCX) [file pntd.0003522.s005.docx]

| **Day since admission** | **WS-** | **WS+/severe** | **Total** |
| --- | --- | --- | --- |
| 0 | 6 | 14 | **20** |
| 1 | 1 | 1 | **2** |
| 2 | 2 | 7 | **9** |
| 3 | 3 | 2 | **5** |
| 4 | 6 | 14 | **20** |
| 5 | 0 | 1 | **1** |
| 6 | 1 | 3 | **4** |
| **Total** | **19** | **42** | **61** |
